# Hemophilia B in a female with intellectual disability caused by a deletion of Xq26.3q28 encompassing the *F9*

**DOI:** 10.1002/mgg3.425

**Published:** 2018-09-27

**Authors:** Sara C. M. Stoof, Rogier Kersseboom, Femke A. T. de Vries, Marieke J. H. A. Kruip, Anneke J. A. Kievit, Frank W. G. Leebeek

**Affiliations:** ^1^ Department of Hematology Erasmus University Medical Centre Rotterdam The Netherlands; ^2^ Department of Clinical Genetics Erasmus University Medical Centre Rotterdam The Netherlands; ^3^ Present address: Medical Service Tragel Zorg Clinge The Netherlands

**Keywords:** *F9*, hemophilia B, intellectual disability, X‐chromosomal deletion

## Abstract

**Background:**

Hemophilia B is an X‐linked recessive disorder caused by mutations in the *F9* on Xq27.1. Mainly males are affected but about 20% of female carriers have clotting factor IX activity below 0.40 IU/ml and bleeding problems. Fragile‐X syndrome (*FMR1*) and FRAXE syndrome (*AFF2*) are well‐known causes of X‐linked recessive intellectual disability. Simultaneous deletion of both *FMR1* and *AFF2* in males results in severe intellectual disability. In females the phenotype is more variable. We report a 19‐year‐old female with severe intellectual disability and a long‐standing bleeding history.

**Methods:**

A SNP array analysis (Illumina Human Cyto 12‐SNP genotyping array) and sequencing of *F9* were performed. Laboratory tests were performed to evaluate the bleeding diathesis.

**Results:**

Our patient was diagnosed with mild hemophilia B after finding an 11 Mb deletion of Xq26.3q28 that included the following genes among others *IDS*,*SOX3*,*FMR1*,*AFF2*, and *F9*.

**Conclusion:**

The case history demonstrates that a severe bleeding tendency suggestive of a hemostasis defect in patients with intellectual disability warrants careful hematological and genetic work‐up even in the absence of a positive family history.

## 1 INTRODUCTION

Hemophilia B is an X‐linked recessive bleeding disorder caused by mutations in the *F9* (OMIM 300746) on Xq27.1 resulting in reduced factor IX (FIX) activity. It occurs in about 2.8 cases per 100.000 males (Berntorp & Shapiro, 2012). Patients may experience surgery or trauma‐induced bleeding and, if more severe, spontaneous bleedings, for which they can be treated prophylactically or on‐demand with FIX concentrate. Although mainly males are affected, about 20% of female carriers have FIX activity below 0.40 IU/ml (Konkle, Josephson, & Nakaya Fletcher, 1993), which is thought to be caused by skewed X‐inactivation. Mutations in the *F9* occur de novo in up to half of the families. Point mutations are the most frequent cause of hemophilia B, deletions and duplications account for only 3% (Li, Miller, Payne, & Craig Hooper, 2013). Approximately 30% of affected males have no family history of hemophilia B (Sommer, Scaringe, & Hill, 2001). Here, we report a 19‐year‐old female with severe intellectual disability (ID) and a long‐standing bleeding history, who was diagnosed with mild hemophilia B after finding a large Xq‐deletion that included the *F9*.

### 1.1 Ethical compliance

This case study was not subject to the Medical Research Involving Human Subjects Act (WMO) as it only involved file research and no additional diagnostic tests were performed. Informed consent of the legal representatives of our patient was obtained.

## 2 METHODS AND RESULTS

The patient was the third child of healthy unrelated parents and had two healthy sisters. There was no bleeding disorder known in the family. She was born at 41 weeks after spontaneous delivery, weighing 3,450 g (50th percentile). Apgar scores were 10 and 10 at 1 and 5 min, respectively. After birth she was hypotonic and had feeding difficulties. She had severe developmental delay already apparent during infancy, severely impaired speech development, and did not walk until the age of 10 years. Her past medical history was remarkable because of a long‐standing history of easy bruising and several severe bleeding episodes. At age 2 she fell from a chair and developed a large head wound. Ten days after initial healing, a rebleeding occurred. At 12 years she fell off the stairs and developed a severe perineal bleed for which she was admitted to the hospital in hypovolemic shock. She received multiple blood transfusions and rebleeding occurred twice with 1‐week interval. She also had a tooth extraction and another head trauma with prolonged bleeding. Occasionally she had large hematomas mostly on the abdomen. No muscle or joint bleeds occurred. No detailed investigation for a bleeding disorder was performed.

**Figure 1:**
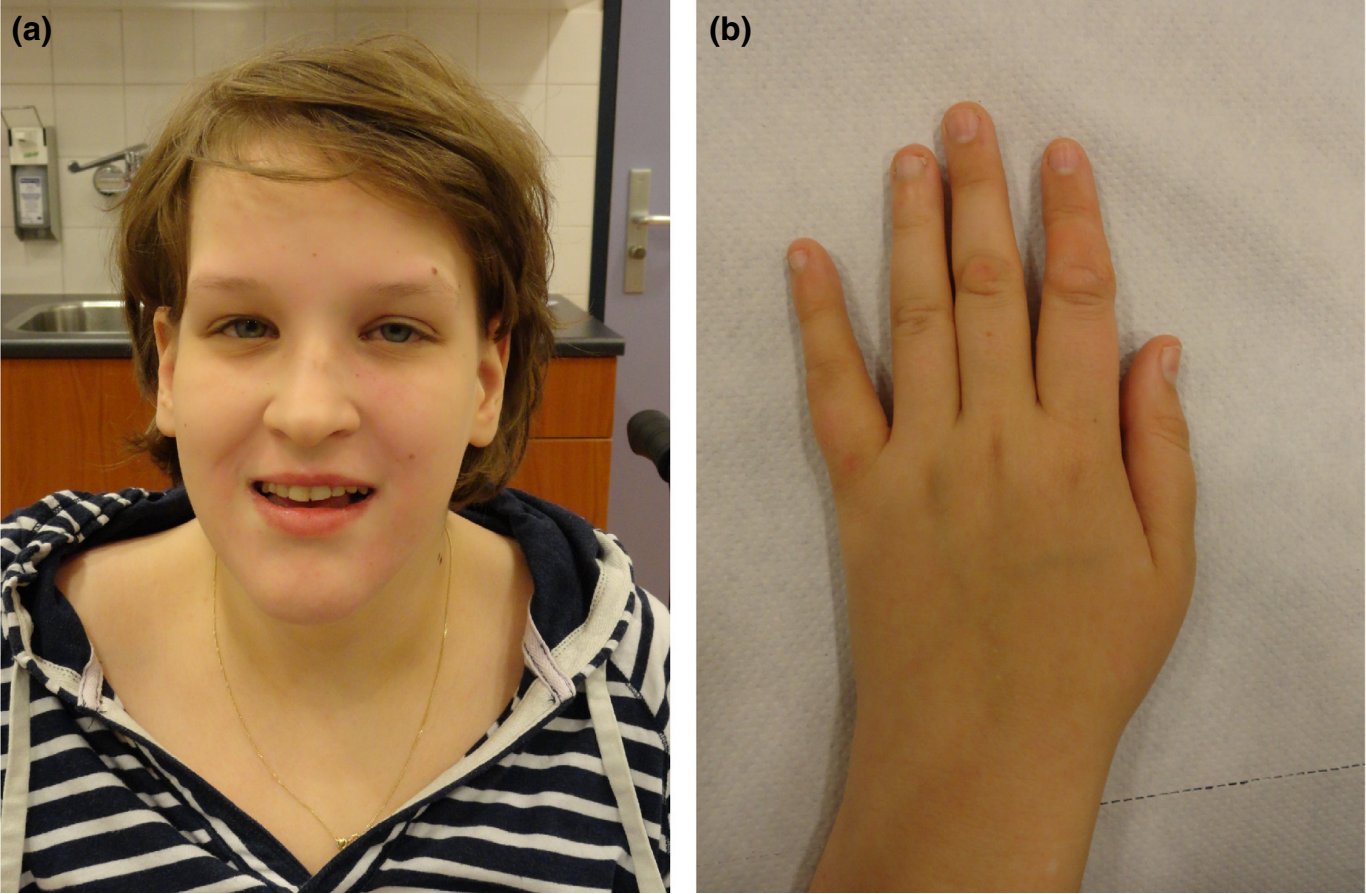
Pictures of the patient. (a) Notice deep set eyes, periorbital fullness, wide nasal root, thin upper vermilion border, and prominent chin. (b) Notice small hands with smooth skin and slight tapering of the fingers

At 19 years of age she was seen at the department of clinical genetics (Figure 1). She had a length of 158 cm (−1.8 *SD*) and a head circumference of 54.6 cm (−0.5 *SD*). She was obese with a good appetite and lack of satiety especially during childhood. Clinical examination revealed abnormal fat distribution mainly localized on the abdomen, thighs and buttocks. She had deep set eyes, periorbital fullness, mild upslanting eyelids, a prominent chin, large ears, small hands 15.7 cm (−3 *SD*), slight tapering of the fingers, soft skin of the hands, joint hyperlaxity of the hands and small feet (Figure 1). On neurological examination she was severely hypotonic, had a stumbling gait and dysarthria. She communicated using few words and gestures. She has intermittent exotropia. Also, she still has urinary and fecal incontinence. She has primary amenorrhea but normal secondary sex characteristics, without signs of premature ovarian failure (POF) on endocrinological evaluation (LH 4.8 U/L; FSH 4.9 U/L; estrogen 93 pmol/L).

**Figure 2:**
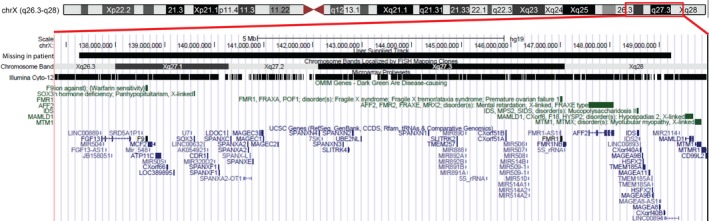
Schematic representation of X‐chromosome and genes in the Xq26‐q28 region. Region of deletion in our patient is highlighted by the red box. The OMIM disease‐associated genes in green and all UCSC annotated genes in blue within the deletion region (hg19) are shown. Image was adapted from http://genome.ucsc.edu/cgi-bin/hgTracks

At 2 years of age, chromosome analysis showed a normal female karyotype. Metabolic blood‐ and urine analyses, including sialotransferrines, were normal. DNA analyses for Prader‐Willi‐ and Fragile‐X‐syndrome were both negative. An MRI of the brain at the age of 3 showed a thin corpus callosum at the splenium, but no other structural abnormalities. Recently, SNP array analysis (Illumina Human Cyto 12‐SNP genotyping array) was performed which showed an 11 Mb interstitial deletion of the chromosomal region Xq26.3q28 [arr Xq26.3q28(137,097,172–148,991,868)]. The region contains 118 annotated genes (Figure 2). The OMIM disease‐associated genes located in this region are *IDS* (OMIM 300823), *SOX3* (OMIM 313430), *FMR1* (OMIM 309550), *AFF2* (OMIM 300806), and *F9* (Figure 2). The Xq26.3q28 deletion was either de novo or due to (germline) mosaicism in one of the parents. FISH analysis in both the patient and her parents did not reveal structural X‐chromosome abnormalities.

Because of the bleeding diathesis and potential FIX defect based on the *F9* deletion, the patient was referred by the clinical geneticist to the hemophilia treatment center. Laboratory results were normal for PFA, VWF:Ag, VWF:CB, VWF:Act, and PT. FIX was 0.20 IU/ml (normal value: 0.60–1.40 IU/ml). Surprisingly, there was a normal APTT (29 s, normal value 22–32 s), most likely due to a FVIII of 1.59 IU/ml (normal value: 0.60–1.40 IU/ml).

She scored a 5 on the ISTH (International Society on Thrombosis and Hemostasis) Bleeding Assessment Tool. Based on her history and the laboratory results she was diagnosed as a hemophilia B carrier due to a *F9* deletion.

To exclude the presence of a second mutation on the other *F9*‐allele contributing to the phenotype, we performed sequence analysis of *F9*. This did not reveal any additional mutations. In rare cases moderate to severe hemophilia B has been seen in females and this has generally been ascribed to skewed X‐inactivation. To test for skewed X‐inactivation we analyzed urine for the presence of abnormal mucopolysaccharide patterns indicative of decreased IDS activity in Hunter syndrome (mucopolysaccharidosis type 2, MPS2). Additionally, we performed pituitary function evaluation (thyroid axis, growth hormone axis, and prolactin) and tested X‐inactivation in peripheral blood leukocytes using the analysis of the methylation status of the AR locus located in Xq12 (Allen, Zoghbi, Moseley, Rosenblatt, & Belmont, 1992). No evidence for MPS2, pituitary dysfunction or skewed X‐inactivation was found.

## 3 DISCUSSION

We report a 19‐year‐old female with severe intellectual disability (ID) and a long‐standing bleeding history, who was diagnosed with mild hemophilia B after finding a large Xq‐deletion that included the *F9*. This demonstrates that a severe bleeding tendency suggestive of a hemostasis defect in patients with intellectual disability warrants careful hematological and genetic work‐up even in the absence of a positive family history. *F9* deletions can range from a few base pairs to several megabases and are found in approximately 3% of patients with hemophilia B. In our patient the hemizygous state of *F9* likely caused mild hemophilia B as is well known in carriers of hemophilia (Nisen & Waber, 1989). In accordance with a previous study, we could not demonstrate skewed X‐inactivation (Orstavik, Scheibel, Ingerslev, & Schwartz, 2000). The inability to consistently demonstrate skewed X‐inactivation in hemophilia B carriers most likely reflects the usage of blood for these studies. A more reliable way to study the role of the X‐inactivation pattern in hemophilia B carriers would be to directly examine liver tissue, which is non‐ethical. Although skewed X‐inactivation is the most important hypothesis to explain variability in FIX activity in carriers, it cannot be excluded that other factors (including genetic) influence factor IX activity in carriers.

Several large deletions of the Xq26‐q28 region have been reported that partly overlap with our patient's deletion. The other OMIM disease‐associated genes located in the deletion found in our patient include *IDS* (mucopolysaccharidosis type 2, MPS2) (Johnson, van Diggelen, Dajnoki, & Bodamer, 2013), *SOX3* (related to pituitary development) (Rizzoti et al., 2004), *FMR1* (Fragile‐X syndrome), and *AFF2* (FRAXE syndrome) (Cordts, Christofolini, Dos Santos, Bianco, & Barbosa, 2011; Moore et al., 1999). A unique feature in our patient was that in addition to the genes *FMR1*,* AFF2*, and *IDS*,* F9* was deleted. Hemizygosity for *IDS* has rarely been shown to result in MPS2 in females (Clarke et al., 1992). In our patient no evidence for MPS2 was found. Duplications of the *SOX3* and polyalanine tract expansions of the C‐terminal domain of *SOX3* have been linked to septo‐optic dysplasia, ID, and panhypopituitarism. In accordance with a previous publication reporting *SOX3* deletion, we found no evidence for pituitary dysfunction or visual problems (Helle et al., 2013). A more recent paper suggested that the deletion of *SOX3* may even be causative of mild intellectual disability (Jourdy et al., 2016). Deletion of *FMR1* and *AFF2* has been shown to cause fragile X syndrome and probably FRAXE‐syndrome (Coffee et al., 2008; Sahoo et al., 2011). Simultaneous deletion of both *FMR1* and *AFF2* in males results in severe ID and variable other features including hypotonia, epilepsy, obesity, and autism (Coffee et al., 2008). In females the phenotype is more variable: some had ID, obesity, and primary amenorrhea, whereas others had primary amenorrhea or POF without ID (Coffee et al., 2008; Mercer et al., 2013). The variability of the phenotype in females may be related to differences in X‐inactivation patterns. This is supported by the finding that within families there may be marked variability in the grade of intellectual ability and age of onset of POF (Fimiani et al., 2006; Mercer et al., 2013). It remains unclear why deletion of the Xq26.3q28 region is involved with POF. One theory is that *FMR1* is involved, because premutations in *FMR1* are associated with POF. It is unclear whether deletion of *FMR1* has a similar effect. An alternative explanation is that other genes within the Xq26.3q28 region are involved.

The case history demonstrates that a severe bleeding tendency suggestive of a hemostasis defect in patients with intellectual disability warrants careful hematological and genetic work‐up even in the absence of a positive family history. Especially, since numerous genetic conditions are characterized by both intellectual disability and increased bleeding tendency and a bleeding disorder may be recognized less easily in people with intellectual disability (as is the case for many medical conditions).

## CONFLICT OF INTEREST

The authors declare no conflicts of interest.

## AUTHOR CONTRIBUTION

RK, FWGL, and JAK clinically reviewed the patient and wrote the manuscript. SCMS performed research and wrote the manuscript. FATV performed analyses and critically revised the manuscript. MJHAK interpreted the data and critically revised the manuscript. All authors approved the final version of the manuscript.

## PATIENT CONSENT

Informed consent of the parents was obtained.
